# Publisher Correction: Wearable device-based health equivalence of different physical activity intensities against mortality, cardiometabolic disease, and cancer

**DOI:** 10.1038/s41467-025-65754-4

**Published:** 2025-10-30

**Authors:** Raaj Kishore Biswas, Matthew N. Ahmadi, Adrian Bauman, Karen Milton, Nicholas A. Koemel, Emmanuel Stamatakis

**Affiliations:** 1 Mackenzie Wearables Research Hub, Charles Perkins Centre, The University of Sydney, Sydney, NSW Australia; 2 School of Health Sciences, Faculty of Medicine and Health, The University of Sydney, Sydney, NSW Australia; 3 School of Public Health, Faculty of Medicine and Health, The University of Sydney, Sydney, NSW Australia; 4 Norwich Medical School, University of East Anglia, Norwich, UK

**Keywords:** Risk factors, Diseases

Correction to: *Nature Communications* 10.1038/s41467-025-63475-2, published online 7 October 2025

In the version of the article initially published, the images presented in Figs. 1 and 2 were interchanged. Figure 1 (top trace, “Type 2 diabetes”) and Fig. 2 (top trace, “PA cancer mortality”) are now amended in the HTML and PDF versions of the article.

