# Supplementary material for: Wearable device-based health equivalence of different physical activity intensities against mortality, cardiometabolic disease, and cancer
Source: Nat Commun. 2025 Oct 7;16:8315. doi: 10.1038/s41467-025-63475-2 (PMC12504536; doi:10.1038/s41467-025-63475-2)
Supplement: Supplementary file 1 — Supplementary Information [file 41467_2025_63475_MOESM1_ESM.pdf]

Supplemental Wearable Device-Based Health Equivalence of different Physical Activity Intensities against mortality, cardiometabolic disease, and cancer

Supplementary Figure 1: Flow diagram of study participants

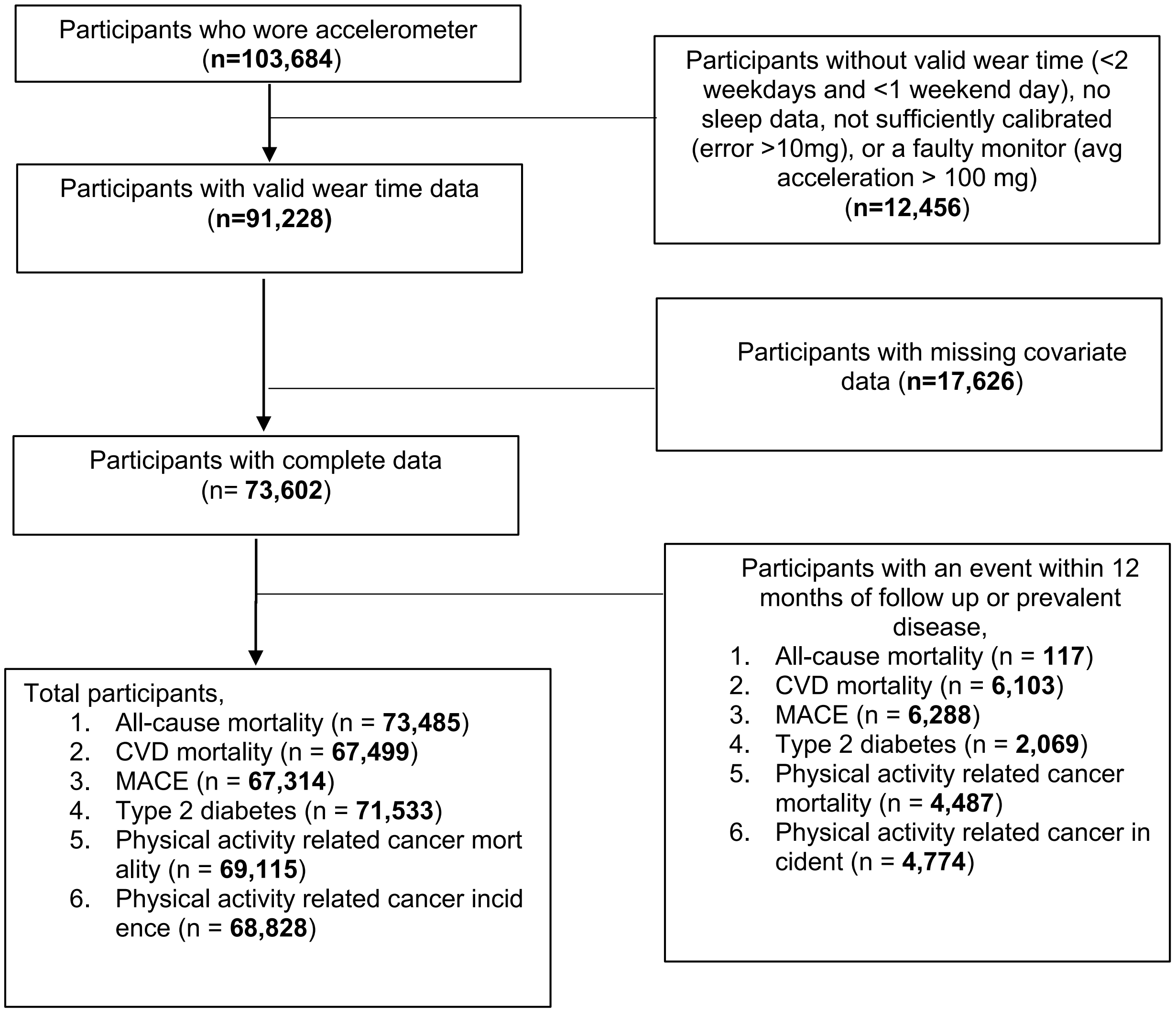

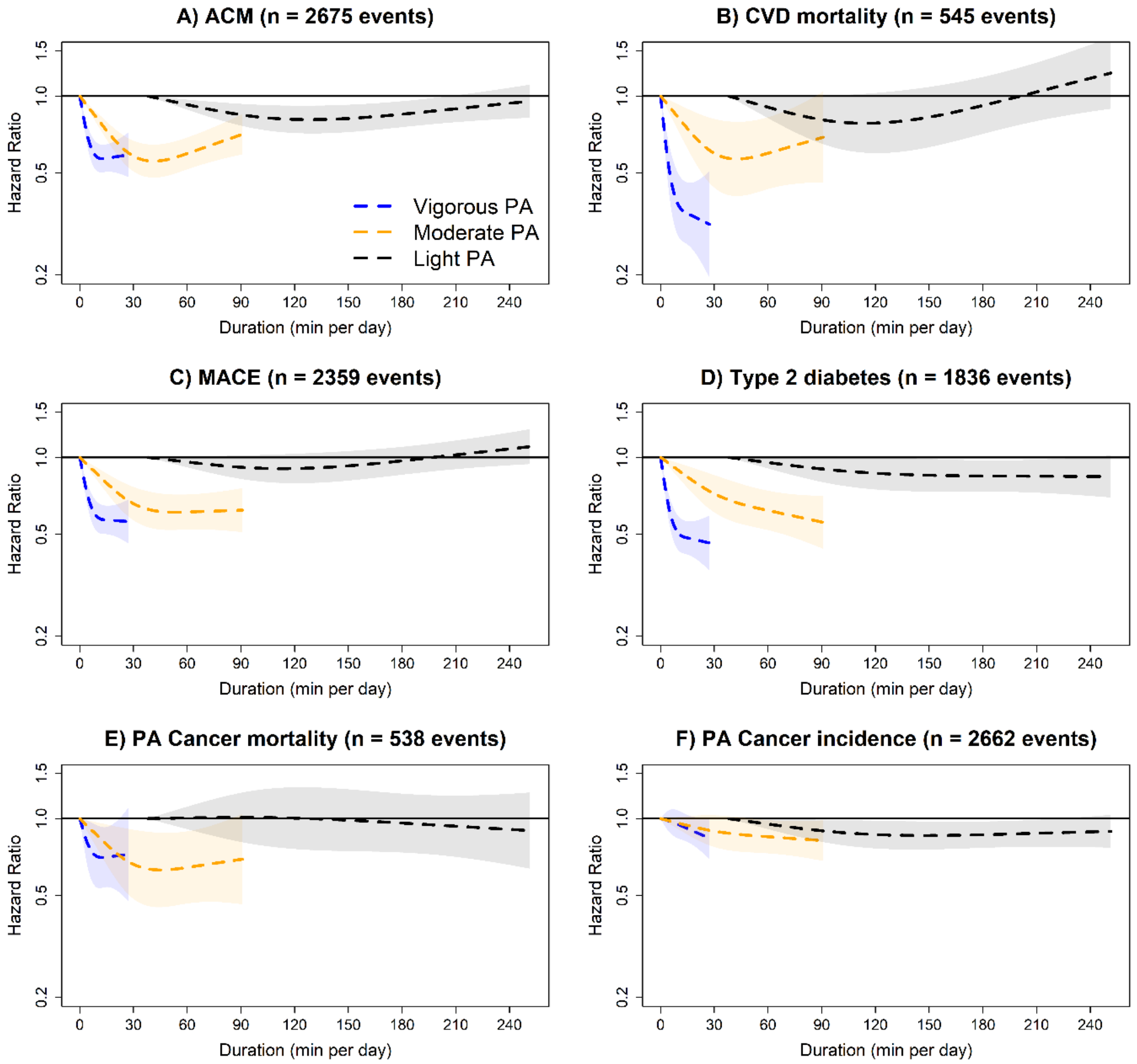

Supplementary Figure 2: Adjusted dose response associations of daily incidental vigorous (VPA), moderate (MPA), and light (LPA) intensity physical activity with all-cause mortality, major adverse cardiovascular event (MACE), CVD mortality, type 2 diabetes, physical activity related cancer mortality and physical activity related cancer incidence. **Panel A:** All-cause mortality (n = 73,485; events = 2,675), **Panel B:** CVD mortality (n = 67,499; events = 545), **Panel C:** MACE (n = 67,314; events = 2,359), **Panel D:** Type 2 diabetes (n = 71,533; events = 1,836), **Panel E:** physical activity related cancer mortality (n = 67,499; events = 538), **Panel F:** physical activity related cancer incidence (n = 68,828; events = 2,662). Analyses were adjusted for sex, age, education, ethnicity, fruit and vegetable consumption, smoking history, alcohol consumption, sleep duration, discretionary screentime, cardiovascular disease (CVD) related medication use (insulin, blood pressure, cholesterol) and family history of cancer and CVD. For ACM and type 2 diabetes, analyses were adjusted for a previous cancer and CVD diagnosis; for MACE and CVD, analyses were adjusted for previous cancer incidence; and for cancer, analyses were adjusted for previous CVD diagnosis. Each physical activity intensity-specific spline model was mutually adjusted for physical activity energy expenditure from other

sintensities estimated using established methods[5]. Referent data point was set to zero minutes for VPA and MPA splines and the minimum value of LPA (37.9 minutes) for LPA splines. All analyses excluded participants who had an event in the first year of follow-up and prevalent major CVD diagnosis at or prior to the accelerometry baseline for MACE and CVD mortality outcomes. Cancer analyses similarly excluded diagnosed cases of cancer at or prior to the accelerometry baseline. For type 2 diabetes previous diagnosed cases at or prior to the accelerometry baseline were excluded.

Supplementary text: Wearable device-based physical activity classification

Physical activity was classified using a previously validated Random Forest (RF) activity classifier.[6] RF is an ensemble of multiple decision trees. Each tree is learned on a bootstrap sample of training data and each node in the tree is split using the best among a randomly selected set of acceleration features. The decisions from each tree are aggregated and a final model prediction is based on majority vote. The RF model requires very little pre-processing of the data, as the features do not need to be normalized. Additionally, the model is resistant to over fitting the training data because each tree within the forest is independently grown to maximum depth using a randomly selected subset of features.

The classifier categorized physical activity in 10 second windows into 1 of 4 activity classes: sedentary, standing utilitarian movements (ironing a shirt, washing dishes), walking activities (gardening, active commuting, mopping floors), running/high energetic activities (active playing with children). These activities were then assigned to 1 of 4 activity intensities: sedentary, light, moderate, and vigorous (**Supplementary Figure 3**). Walking activities were classified as light (<100mg), moderate (≥100mg) and vigorous (≥400mg) intensity. The diagram in **Supplementary Figure 3** depicts how activity intensity was classified Differentiation from sleep[7] and non-wear[8] was identified using the change in tilt angle and acceleration standard deviation. Monitors were calibrated[9] and corrected for orientation[10] using previously published methods.

Supplementary Figure 3: Physical activity type and intensity diagram

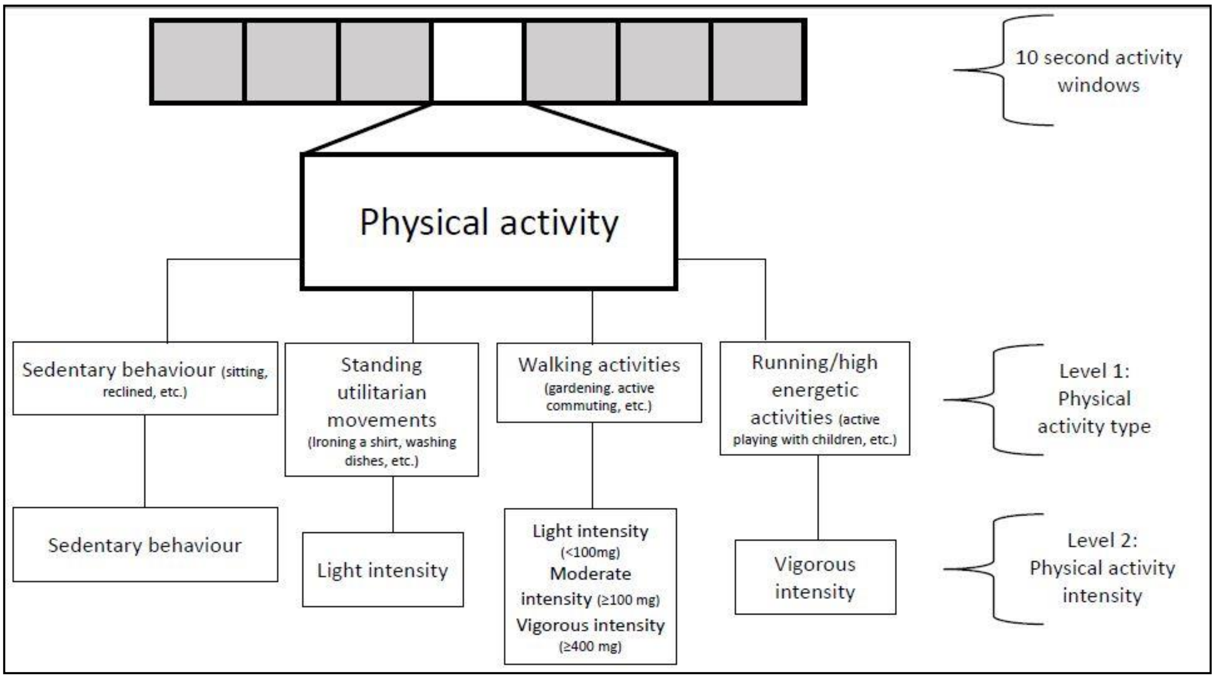

Activities in an independent sample of 98 participants (Age = 56.4 ± 15.7; 53.1% female) from the US[11] (University of California Irvine Center for Machine Learning and Intelligent Systems *Physical Activity Monitoring for Aging People* study [published data], accessible at https://archive.ics.uci.edu/ml/datasets) and Australia[12] (University of Queensland *Where and When at Work* study [published data], and University of Sydney *Intermittent Lifestyle Physical Activity* Study [unpublished data]) providing 103,607 activity samples from structured and free-living activities (17,267 minutes) were used to assess robustness and generalizability of the classifier. For free-living activities participant-worn or researcher-held Go-Pro video-recordings were used to attain ground-truth physical activity. Video files were imported into the Noldus Observer XT software for continuous direct observation coding. A two-stage direct observation scheme was implemented in which the participant's movement behaviour was coded for activity type and then activity intensity based on Compendium of Physical Activities[13]. The direct observation system generated a vector of date-time stamps corresponding to the start and finish of each movement event, which were used to assign the activity codes to the corresponding time segments of the accelerometer data. Interobserver reliability was assessed by dual coding (**Supplementary Table 7-8**). The intraclass correlation coefficient for coding activities was 0.912 (0.866-0.942).

Performance was further evaluated in a separate sample of 151 adults (age range 18-91 years, 65.6% female) recruited from the UK[14] (University of Oxford *Capture 24* study [published data], accessible at https://ora.ox.ac.uk/objects/uuid:99d7c092-d865-4a19-b096-cc16440cd001). Participants in this dataset wore body cameras that provided pictures every 20 seconds to annotate ground-truth free-living activity labels (**Supplementary Figure 4)**. The picture-based activity coding scheme has been previously described[9]. A total of 172,360 activity samples (28,727 minutes) were provided by participants.

Supplementary Table 7: Intensity classification performance in n=102 US and Australian adults (exercisers and non-exercisers pooled; age = 55.8 ± 12.4; 55.8% female) providing 105,767 activity samples from structured (exercised-based) and free-living activities (17,627 minutes) (from published and unpublished data).

	Sensitivity	Specificity	Precision	F-score	Overall Accuracy	Weighted Kappa	Overall F-score
Sedentary	86.5	93.7	90.5	88.5			
Light	71.2	89.4	55.8	62.6			
Moderate	85.4	96.6	92.7	88.9			
Vigorous	95.4	99.4	94.6	95.0			
					84.6	0.78	83.8

Supplementary Table 8: Confusion matrix of incidental behaviour, among participants who reported as non-exercisers in the independent validity testing (n=82; 3688 minutes of activity data)

**Predictions**	**Ground Truth**			
	Sedentary	Light	Moderate	Vigorous
Sedentary	**92.4%**	7.6%	-	-
Light	13.3%	**80.8%**	5.9%	-
Moderate	-	11.7%	**88.1%**	0.2%
Vigorous	-	1.2%	1.5%	**97.3%**

Rows= ground truth; columns=predictions; bold=correct labels; numbers represent each 10-second window; Derived from the US and Australian datasets

Supplementary Figure 4: Participant-level specific recall and precision in the UK sample

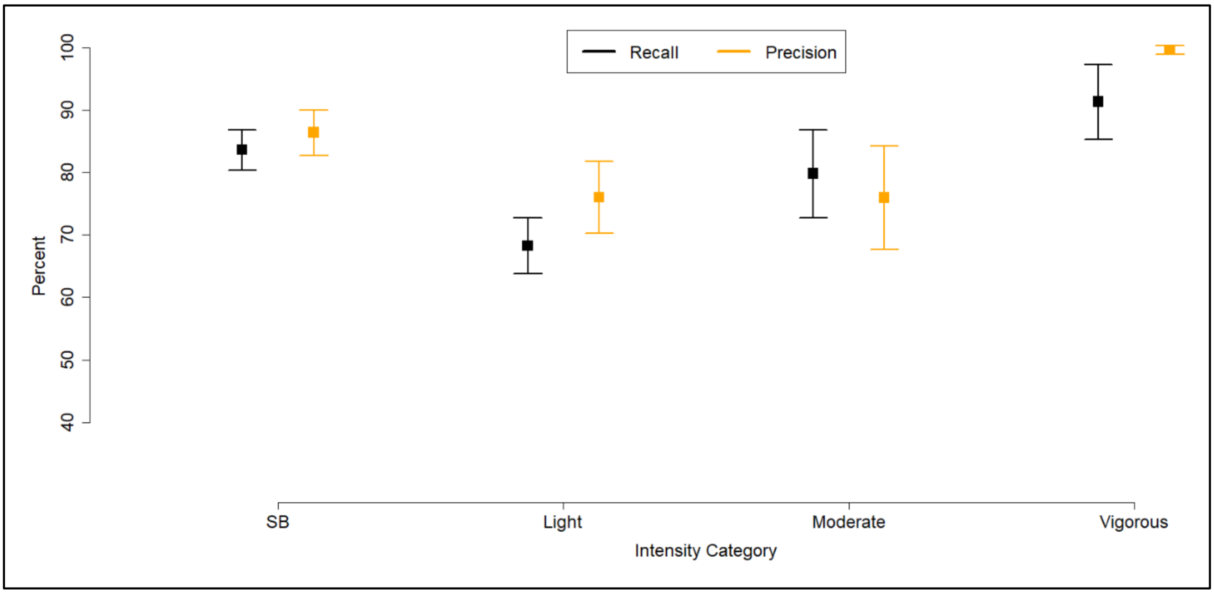

Supplementary References

1 Stamatakis, E. *et al.* Association of wearable device-measured vigorous intermittent lifestyle physical activity with mortality. *Nat Med* **28**, 2521-2529 (2022). https://doi.org/10.1038/s41591-022-02100-x

2 Ahmadi, M. N. *et al.* Brief bouts of device-measured intermittent lifestyle physical activity and its association with major adverse cardiovascular events and mortality in people who do not exercise: a prospective cohort study. *The Lancet Public Health* **8**, e800-e810 (2023).

3 Boonpor, J. *et al.* Types of diet, obesity, and incident type 2 diabetes: Findings from the UK Biobank prospective cohort study. *Diabetes Obes Metab* **24**, 1351-1359 (2022). https://doi.org/10.1111/dom.14711

4 Moore, S. C. *et al.* Association of Leisure-Time Physical Activity With Risk of 26 Types of Cancer in 1.44 Million Adults. *JAMA Intern Med* **176**, 816-825 (2016). https://doi.org/10.1001/jamainternmed.2016.1548

5 White, T. *et al.* Estimating energy expenditure from wrist and thigh accelerometry in free-living adults: a doubly labelled water study. *International Journal of Obesity* **43**, 2333-2342 (2019). https://doi.org/10.1038/s41366-019-0352-x

6 Pavey, T. G., Gilson, N. D., Gomersall, S. R., Clark, B. & Trost, S. G. Field evaluation of a random forest activity classifier for wrist-worn accelerometer data. *J. Sci. Med. Sport* **20**, 75-80 (2017). https://doi.org/10.1016/j.jsams.2016.06.003

7 van Hees, V. T. *et al.* Estimating sleep parameters using an accelerometer without sleep diary. *Sci. Rep.* **8**, 12975 (2018). https://doi.org/10.1038/s41598-018-31266-z

8 Ahmadi, M. N., Nathan, N., Sutherland, R., Wolfenden, L. & Trost, S. G. Non-wear or sleep? Evaluation of five non-wear detection algorithms for raw accelerometer data. *J. Sports Sci.* **38**, 399-404 (2020). https://doi.org/10.1080/02640414.2019.1703301

9 Sipos, M., Paces, P., Rohac, J. & Novacek, P. Analyses of Triaxial Accelerometer Calibration Algorithms. *IEEE Sens. J.* **12**, 1157-1165 (2012). https://doi.org/10.1109/jsen.2011.2167319

10 Mizell, D. in *Proceedings of the 7th IEEE International Symposium on Wearable Computers* (White Plains, NY).

11 Reiss, A., Weber, M. & Stricker, D. in *2011 IEEE International Conference on Systems, Man, and Cybernetics* (IEEE, 2011).

12 Clark, B., Winker, E., Ahmadi, M. & Trost, S. Comparison of three algorithms using thigh-worn accelerometers for classifying sitting, standing, and stepping in free-living office workers. *J. Meas. Phys. Behav.* **4**, 89-95 (2021). https://doi.org/10.1123/jmpb.2020-0019

13 Ainsworth, B. E. *et al.* 2011 compendium of physical activities. *Med. Sci. Sports Exerc.* **43**, 1575-1581 (2011). https://doi.org/10.1249/mss.0b013e31821ece12

14 Willetts, M., Hollowell, S., Aslett, L., Holmes, C. & Doherty, A. Statistical machine learning of sleep and physical activity phenotypes from sensor data in 96,220 UK Biobank participants. *Sci Rep* **8** (2018).
