# Supplementary material for: Wearable device-based health equivalence of different physical activity intensities against mortality, cardiometabolic disease, and cancer
Source: Nat Commun. 2025 Oct 7;16:8315. doi: 10.1038/s41467-025-63475-2 (PMC12504536; doi:10.1038/s41467-025-63475-2)
Supplement: Supplementary file 2 — Supplementary Data 1 [file 41467_2025_63475_MOESM2_ESM.docx]

**Supplementary Table 1:** Participant baseline characteristics by VPA duration (n= 73,485).

| **Tertiles of VPA duration (min/day)** | $\boldsymbol{0}$ | $\boldsymbol{\geq0-2.5}$ | $\boldsymbol{2.5-6.5}$ | $\boldsymbol{\geq6.5}$ | **Overall** |
| --- | --- | --- | --- | --- | --- |
| **Sample size (n)** | 1,604 | 23,961 | 23,960 | 23,960 |  |
| **Follow up (in years),**  **mean (SD)** | 7.8 (1.2) | 7.9 (1.0) | 8.0 (0.9) | 8.0 (0.8) | 8.0 (0.9) |
| **Age, mean (SD)** | 64.3 (7.1) | 63.3 (7.6) | 61.6 (7.7) | 59.6 (7.8) | 61.6 (7.9) |
| **Male** | 491 (30.6) | 8,882 (37.1) | 10,410 (43.4) | 12,282 (51.3) | 32,065 (43.6) |
| **Ethnicity - White** | 1,552 (96.8) | 23,103 (96.4) | 23,065 (96.3) | 22,940 (95.7) | 70,660 (96.2) |
| **Smoking history** | | | | | |
| **Current** | 200 (12.5) | 1,672 (7.0) | 1,508 (6.3) | 1,472 (6.1) | 4,852 (6.6) |
| **Never** | 859 (53.6) | 13,449 (56.1) | 13,743 (57.4) | 14,351 (59.9) | 42,402 (57.7) |
| **Previous** | 545 (34.0) | 8,840 (36.9) | 8,709 (36.3) | 8,137 (34.0) | 26,231 (35.7) |
| **Alcohol consumption^1^** | | | | | |
| **Never** | 66 (4.1) | 735 (3.1) | 586 (2.4) | 558 (2.3) | 1,945 (2.6) |
| **Ex-drinker** | 472 (29.4) | 8,345 (34.8) | 9,276 (38.7) | 9,573 (40.0) | 27,666 (37.6) |
| **Within guidelines** | 999 (62.3) | 14,208 (59.3) | 13,528 (56.5) | 13,303 (55.5) | 42,038 (57.2) |
| **Above guidelines** | 472 (29.4) | 8,345 (34.8) | 9,276 (38.7) | 9,573 (40.0) | 27,666 (37.6) |
| **Education** | | | | | |
| **College** | 616 (38.4) | 10,453 (43.6) | 10,652 (44.5) | 11,016 (46.0) | 32,737 (44.5) |
| **A/AS level** | 224 (14.0) | 3,073 (12.8) | 3,253 (13.6) | 3,206 (13.4) | 9,756 (13.3) |
| **O level** | 76 (4.7) | 1,250 (5.2) | 1,211 (5.1) | 1,284 (5.4) | 3,821 (5.2) |
| **CSE** | 53 (3.3) | 809 (3.4) | 926 (3.9) | 1,073 (4.5) | 2,861 (3.9) |
| **NVQ/HND/HNC** | 76 (4.7) | 1,250 (5.2) | 1,211 (5.1) | 1,284 (5.4) | 3,821 (5.2) |
| **Other** | 298 (18.6) | 3,538 (14.8) | 3,091 (12.9) | 2,721 (11.4) | 9,648 (13.1) |
| **Fruit and vegetable consumption (serving/day)^2^** | 7.5 (4.2) | 8.0 (4.4) | 8.1 (4.3) | 8.0 (4.4) | 8.0 (4.4) |
| **Medication** | | | | | |
| **Cholesterol** | 334 (20.8) | 4,262 (17.8) | 3,193 (13.3) | 2,228 (9.3) | 10,017 (13.6) |
| **Insulin** | 23 (1.4) | 152 (0.6) | 104 (0.4) | 96 (0.4) | 375 (0.5) |
| **Blood pressure** | 406 (25.3) | 4,296 (17.9) | 3,249 (13.6) | 2,357 (9.8) | 10,308 (14.0) |
| **Diagnosed CVD** | 45 (2.8) | 2,876 (12.0) | 1,980 (8.3) | 1,085 (4.5) | 5,986 (8.1) |
| **Diagnosed cancer** | 132 (8.2) | 1,715 (7.2) | 1,432 (6.0) | 1,091 (4.6) | 4,370 (5.9) |
| **Family history of CVD** | 932 (58.1) | 13,833 (57.7) | 13,148 (54.9) | 12,504 (52.2) | 40,417 (55.0) |
| **Family history of Cancer** | 504 (31.4) | 6,839 (28.5) | 6,704 (28.0) | 6,553 (27.3) | 20,600 (28.0) |
| **Light activity (min/day), mean (SD)** | 89.7 (53.1) | 108.8 (57.0) | 116.0 (58.3) | 116.3 (56.4) | 113.2 (57.4) |
| **Moderate activity (min/day),**  **mean (SD)** | 13.6 (16.0) | 22.3 (17.8) | 33.3 (20.3) | 41.9 (22.2) | 32.1 (21.8) |
| **Sleep duration (min/day),**  **mean (SD)** | 494.0 (248.6) | 439.4 (93.4) | 442.1 (79.8) | 442.0 (72.4) | 442.3 (89.7) |
| **Discretionary screentime (min/day), mean (SD)** | 4.3 (2.3) | 4.0 (2.1) | 3.8 (2.0) | 3.6 (2.0) | 3.8 (2.1) |
| **ACM** | 131 (8.2) | 1,130 (4.7) | 829 (3.5) | 585 (2.4) | 2,675 (3.6) |
| **CVD mortality** | 33 (2.1) | 241 (1.1) | 173 (0.8) | 102 (0.4) | 549 (0.8) |
| **MACE incidence** | 117 (7.3) | 1,834 (7.7) | 1,319 (5.5) | 880 (3.7) | 4,150 (5.6) |
| **Type 2 diabetes** | 188 (11.7) | 1,585 (6.6) | 956 (4.0) | 598 (2.5) | 3,327 (4.5) |
| **PA Cancer mortality** | 26 (1.6) | 281 (1.2) | 243 (1.0) | 154 (0.6) | 704 (1.0) |
| **PA Cancer incidence** | 119 (7.4) | 1,389 (5.8) | 1,169 (4.9) | 912 (3.8) | 3,589 (4.9) |

The columns correspond to duration of VPA. Values represent n (%) unless specified otherwise. ^1^Alcohol consumption: above guidelines are >14 units per week, where 1 unit = 8 g of ethanol. ^2^Fruits and vegetable consumption is servings per day.
