# Supplementary material for: Wearable device-based health equivalence of different physical activity intensities against mortality, cardiometabolic disease, and cancer
Source: Nat Commun. 2025 Oct 7;16:8315. doi: 10.1038/s41467-025-63475-2 (PMC12504536; doi:10.1038/s41467-025-63475-2)
Supplement: Supplementary file 3 — Supplementary Data 2 [file 41467_2025_63475_MOESM3_ESM.docx]

**Supplementary Table 2:** Hazard ratios (HR) associated with 5% increments risk reduction for each physical activity intensity for cardiometabolic outcomes, including cardiovascular (CVD) mortality, major adverse cardiovascular events (MACE) and type 2 diabetes.

| CVD mortality | | | | | | |
| --- | --- | --- | --- | --- | --- | --- |
| Risk reduction (%) | HR | VPA | MPA | LPA | MPA equivalence per 1 minute of VPA | LPA equivalence per 1 minute of VPA |
| 5 | 0.95 | 0.34  (0.33-0.36) | 2.62  (2.58-2.66) | 48.13  (48.10-48.19) | 7.71 (7.17-8.06) | 141.56  (137.43-146.03) |
| 10 | 0.90 | 0.69  (0.68-0.70) | 5.38  (5.34-5.42) | 60.03  (60.00-60.10) | 7.80  (7.63-7.97) | 87.00  (85.71-89.70) |
| 15 | 0.85 | 1.06  (1.05-1.08) | 8.30  (8.25-8.34) | 73.43  (73.39-73.50) | 7.79  (7.64-7.94) | 69.27  (67.95-70.00) |
| 20 | 0.80 | 1.47  (1.45-1.48) | 11.39  (11.34-11.43) | 91.75  (91.69-91.84) | 7.77  (7.66-7.88) | 62.41  (61.95-63.34) |
| 25 | 0.75 | 1.90  (1.88-1.92) | 14.73  (14.68-14.78) | - | 7.75  (7.65-7.86) | - |
| 30 | 0.70 | 2.37  (2.35-2.38) | 18.49  (18.44-18.54) | - | 7.82  (7.75-7.89) | - |
| 35 | 0.65 | 2.89  (2.87-2.91) | 64.74  (62.43-66.89) | - | 22.40  (21.45-23.31) | - |
|  |  |  |  |  | **Median equivalence: 7.78 (7.66-7.97)** | **Median equivalence: 72.47 (71.68-73.73)** |
| MACE | | | | | | |
| Risk reduction (%) | HR | VPA | MPA | LPA | MPA equivalence per 1 minute of VPA | LPA equivalence per 1 minute of VPA |
| 5 | 0.95 | 0.60  (0.58-0.62) | 3.31  (3.26-3.35) | 62.36  (62.30-62.45) | 5.51  (5.26-5.78) | 103.93 (100.48-107.67) |
| 10 | 0.90 | 1.23  (1.21-1.25) | 6.80  (6.75-6.84) | 104.11 (104.04-104.21) | 5.52  (5.40-5.65) | 84.64  (83.23-86.12) |
| 15 | 0.85 | 1.92  (1.90-1.94) | 10.49  (10.43-10.54) | - | 5.48  (5.38-5.55) | - |
| 20 | 0.80 | 2.66  (2.64-2.68) | 14.43  (14.37-14.48) | - | 5.42  (5.36-5.48) | - |
| 25 | 0.75 | 3.52  (3.50-3.54) | 18.82  (18.76-18.87) | - | 5.35  (5.30-5.39) | - |
| 30 | 0.70 | 4.58  (4.55-4.61) | 24.11  (24.04-24.17) | - | 5.27  (5.21-5.31) | - |
| 35 | 0.65 | 6.03  (6.00-6.07) | 31.83  (31.75-31.90) | - | 5.28  (5.23-5.32) | - |
|  |  |  |  |  | **Median equivalence: 5.44 (5.34-5.48)** | **Median equivalence: 86.13 (84.39-87.98)** |
| Type 2 diabetes | | | | | | |
| Risk reduction (%) | HR | VPA | MPA | LPA | MPA equivalence per 1 minute of VPA | LPA equivalence per 1 minute of VPA |
| 5 | 0.95 | 0.48  (0.46-0.49) | 4.36  (4.30-4.41) | 59.82  (59.79-59.88) | 9.18 (8.78-9.59) | 124.63  (122.02-130.17) |
| 10 | 0.90 | 0.97  (0.95-0.99) | 8.96  (8.90-9.01) | 87.39  (87.36-87.46) | 9.23 (8.99-9.48) | 90.09  (88.24-92.06) |
| 15 | 0.85 | 1.50  (1.48-1.52) | 13.83  (13.77-13.89) | 140.35  (140.22-140.50) | 9.22 (9.06-9.39) | 93.57  (92.25-94.93) |
| 20 | 0.80 | 2.07  (2.04-2.08) | 19.18  (19.12-19.24) | - | 9.29 (9.19-9.43) | - |
| 25 | 0.75 | 2.70  (2.68-2.72) | 25.47  (25.40-25.53) | - | 9.43 (9.34-9.53) | - |
| 30 | 0.70 | 3.43  (3.40-3.45) | 33.95  (33.87-34.03) | - | 9.91 (9.82-10.01) | - |
| 35 | 0.65 | 4.30  (4.27-4.32) | 47.54  (47.43-47.63) | - | 11.07 (10.98-11.15) | - |
|  |  |  |  |  | **Median equivalence: 9.40 (9.31-9.60)** | **Median equivalence: 94.03 (92.27-95.30)** |

Risk reduction, based on the hazard ratios (HR) of the dose-response curves presented in Supplementary Figure 2. The values inside the parentheses represent 95% confidence intervals (CI).

HR: hazard ratio; VPA: vigorous physical activity; MPA: moderate physical activity; LPA: light physical activity.
