# Supplementary material for: Wearable device-based health equivalence of different physical activity intensities against mortality, cardiometabolic disease, and cancer
Source: Nat Commun. 2025 Oct 7;16:8315. doi: 10.1038/s41467-025-63475-2 (PMC12504536; doi:10.1038/s41467-025-63475-2)
Supplement: Supplementary file 4 — Supplementary Data 3 [file 41467_2025_63475_MOESM4_ESM.docx]

**Supplementary Table 3:** Hazard ratios (HR) associated with 5% increments risk reduction for each physical activity intensity for all-cause mortality (ACM) and physical activity related cancer outcomes.

| All-cause mortality | | | | | | |
| --- | --- | --- | --- | --- | --- | --- |
| Risk reduction (%) | HR | VPA | MPA | LPA | MPA minutes equivalence per 1 minute of VPA | LPA minutes equivalence per 1 minute of VPA |
| 5 | 0.95 | 0.56  (0.54-0.58) | 2.22  (2.21-2.25) | 51.19 (51.15-51.27) | 3.96  (3.83-4.17) | 91.41  (88.19-94.94) |
| 10 | 0.90 | 1.15  (1.13-1.17) | 4.80  (4.80-4.83) | 66.81 (66.76-66.88) | 4.17  (4.10-4.27) | 58.10  (57.06-59.19) |
| 15 | 0.85 | 1.78  (1.76-1.80) | 7.53  (7.52-7.56) | 85.61 (85.55-85.69) | 4.23  (4.18-4.30) | 48.10  (47.53-48.69) |
| 20 | 0.80 | 2.48  (2.46-2.50) | 10.43  (10.43-10.46) | - | 4.21  (4.17-4.25) | - |
| 25 | 0.75 | 3.27  (3.25-3.29) | 13.53  (13.51-13.56) | - | 4.14  (4.11-4.17) | - |
| 30 | 0.70 | 4.24  (4.21-4.26) | 16.98  (16.94-17.01) | - | 4.01  (3.99-4.04) | - |
| 35 | 0.65 | 5.54  (5.51-5.58) | 21.04  (21.01-21.07) | - | 3.79  (3.77-3.82) | - |
|  |  |  |  |  | **Median equivalence: 4.09 (4.05-4.17)** | **Median equivalence: 52.65 (51.87-53.47)** |
| Physical activity related cancer mortality | | | | | | |
| Risk reduction (%) | HR | VPA | MPA | LPA | MPA equivalence per 1 minute of VPA | LPA equivalence per 1 minute of VPA |
| 5 | 0.95 | 0.90  (0.88-0.93) | 3.29  (3.24-3.33) | 186.13  (186.07-186.21) | 3.65  (3.48-3.78) | 206.81  (200.08-211.60) |
| 10 | 0.90 | 1.87  (1.84-1.89) | 6.75  (6.71-6.80) | 242.92  (242.88-242.99) | 3.62  (3.55-3.70) | 130.60  (128.51-132.06) |
| 15 | 0.85 | 2.94  (2.91-2.97) | 10.41  (10.36-10.46) | - | 3.54  (3.49-3.59) | - |
| 20 | 0.80 | 4.27  (4.24-4.30) | 14.34  (14.28-14.38) | - | 3.36  (3.32-3.39) | - |
| 25 | 0.75 | 6.26  (6.22-6.30) | 18.75  (18.69-18.80) | - | 2.99  (2.97-3.02) | - |
| 30 | 0.70 | 12.29  (12.24-12.34) | 24.17  (24.10-24.23) | - | 1.97  (1.95-1.98) | - |
| 35 | 0.65 | - | 54.26  (53.36-55.16) | - | - | - |
|  |  |  |  |  | **Median equivalence: 3.47 (3.42-3.51)** | **Median equivalence: 156.23 (153.33-159.28)** |
| Physical activity related cancer incidence | | | | | | |
| Risk reduction (%) | HR | VPA | MPA | LPA | MPA equivalence per 1 minute of VPA | LPA equivalence per 1 minute of VPA |
| 5 | 0.95 | 9.60  (9.52-9.67) | 12.61  (12.52-12.71) | 58.57 (58.54-58.63) | 1.31  (1.29-1.34) | 6.10  (6.05-6.16) |
| 10 | 0.90 | 17.28  (17.21-17.35) | 27.43  (27.33-27.54) | 84.64 (84.61-84.71) | 1.59  (1.58-1.60) | 4.90  (4.88-4.92) |
| 15 | 0.85 | 25.29  (25.21-25.36) | 59.38  (59.19-59.56) | - | 2.35  (2.33-2.36) | - |
| 20 | 0.80 | - | - | - | - | - |
| 25 | 0.75 | - | - | - | - | - |
| 30 | 0.70 | - | - | - | - | - |
| 35 | 0.65 | - | - | - | - | - |
|  |  |  |  |  | **Median equivalence: 1.63 (1.62-1.64)** | **Median equivalence: 5.09 (5.06-5.12)** |

Risk reduction, based on the hazard ratios (HR) of the dose-response curves presented in Supplementary Figure 2. The values inside the parentheses represent 95% confidence intervals (CI).

HR: hazard ratio; VPA: vigorous physical activity; MPA: moderate physical activity; LPA: light physical activity.
