# Supplementary material for: Wearable device-based health equivalence of different physical activity intensities against mortality, cardiometabolic disease, and cancer
Source: Nat Commun. 2025 Oct 7;16:8315. doi: 10.1038/s41467-025-63475-2 (PMC12504536; doi:10.1038/s41467-025-63475-2)
Supplement: Supplementary file 5 — Supplementary Data 4 [file 41467_2025_63475_MOESM5_ESM.docx]

**Supplementary Table 4:** STROBE Statement

|  | Item No | Recommendation | | Page  No |
| --- | --- | --- | --- | --- |
| **Title and abstract** | 1 | (*a*) Indicate the study’s design with a commonly used term in the title or the abstract | | 1 |
|  |  | (*b*) Provide in the abstract an informative and balanced summary of what was done and what was found | | 2 |
| Introduction | | | | |
| Background/rationale | 2 | Explain the scientific background and rationale for the investigation being reported | | 3-4 |
| Objectives | 3 | State specific objectives, including any prespecified hypotheses | | 4 |
| Methods | | | | |
| Study design | 4 | Present key elements of study design early in the paper | | 5 |
| Setting | 5 | Describe the setting, locations, and relevant dates, including periods of recruitment, exposure, follow-up, and data collection | | 5-6 |
| Participants | 6 | (*a*) *Cohort study*—Give the eligibility criteria, and the sources and methods of selection of participants. Describe methods of follow-up  *Case-control study*—Give the eligibility criteria, and the sources and methods of case ascertainment and control selection. Give the rationale for the choice of cases and controls  *Cross-sectional study*—Give the eligibility criteria, and the sources and methods of selection of participants | | 10-11 |
|  |  | (*b*) *Cohort study*—For matched studies, give matching criteria and number of exposed and unexposed  *Case-control study*—For matched studies, give matching criteria and the number of controls per case | | NA |
| Variables | 7 | Clearly define all outcomes, exposures, predictors, potential confounders, and effect modifiers. Give diagnostic criteria, if applicable | | 11-12 |
| Data sources/ measurement | 8* | For each variable of interest, give sources of data and details of methods of assessment (measurement). Describe comparability of assessment methods if there is more than one group | | 13 |
| Bias | 9 | Describe any efforts to address potential sources of bias | | 12-13 |
| Study size | 10 | Explain how the study size was arrived at | | 5 |
| Quantitative variables | 11 | Explain how quantitative variables were handled in the analyses. If applicable, describe which groupings were chosen and why | | 11-12 |
| Statistical methods | 12 | (*a*) Describe all statistical methods, including those used to control for confounding | | 12-14 |
|  |  | (*b*) Describe any methods used to examine subgroups and interactions | | NA |
|  |  | (*c*) Explain how missing data were addressed | | 11 |
|  |  | (*d*) *Cohort study*—If applicable, explain how loss to follow-up was addressed  *Case-control study*—If applicable, explain how matching of cases and controls was addressed  *Cross-sectional study*—If applicable, describe analytical methods taking account of sampling strategy | | NA |
|  |  | (*e*) Describe any sensitivity analyses | | NA |
| **Results** |  | |  |  |
| Participants | 13* | | (a) Report numbers of individuals at each stage of study—eg numbers potentially eligible, examined for eligibility, confirmed eligible, included in the study, completing follow-up, and analysed | Supplementary Figure 1 |
|  |  | | (b) Give reasons for non-participation at each stage | Supplementary Figure 1 |
|  |  | | (c) Consider use of a flow diagram | Supplementary Figure 1 |
| Descriptive data | 14* | | (a) Give characteristics of study participants (eg demographic, clinical, social) and information on exposures and potential confounders | Supplementary Table 1 |
|  |  | | (b) Indicate number of participants with missing data for each variable of interest | Supplementary Figure 1 |
|  |  | | (c) *Cohort study*—Summarise follow-up time (eg, average and total amount) | 5 |
| Outcome data | 15* | | *Cohort study*—Report numbers of outcome events or summary measures over time | 5 |
|  |  | | *Case-control study—*Report numbers in each exposure category, or summary measures of exposure | NA |
|  |  | | *Cross-sectional study—*Report numbers of outcome events or summary measures | NA |
| Main results | 16 | | (*a*) Give unadjusted estimates and, if applicable, confounder-adjusted estimates and their precision (eg, 95% confidence interval). Make clear which confounders were adjusted for and why they were included | 5-6, Supplementary Figure 2 |
|  |  | | (*b*) Report category boundaries when continuous variables were categorized | NA |
|  |  | | (*c*) If relevant, consider translating estimates of relative risk into absolute risk for a meaningful time period | NA |
| Other analyses | 17 | | Report other analyses done—eg analyses of subgroups and interactions, and sensitivity analyses | NA |
| **Discussion** |  | |  |  |
| Key results | 18 | | Summarise key results with reference to study objectives | 6-8 |
| Limitations | 19 | | Discuss limitations of the study, taking into account sources of potential bias or imprecision. Discuss both direction and magnitude of any potential bias | 9 |
| Interpretation | 20 | | Give a cautious overall interpretation of results considering objectives, limitations, multiplicity of analyses, results from similar studies, and other relevant evidence | 9 |
| Generalisability | 21 | | Discuss the generalisability (external validity) of the study results | 7-8 |
| **Other information** |  | |  |  |
| Funding | 22 | | Give the source of funding and the role of the funders for the present study and, if applicable, for the original study on which the present article is based | 14-15 |
