# Supplementary material for: Wearable device-based health equivalence of different physical activity intensities against mortality, cardiometabolic disease, and cancer
Source: Nat Commun. 2025 Oct 7;16:8315. doi: 10.1038/s41467-025-63475-2 (PMC12504536; doi:10.1038/s41467-025-63475-2)
Supplement: Supplementary file 6 — Supplementary Data 5 [file 41467_2025_63475_MOESM6_ESM.docx]

**Supplementary Table 5**: Assessment of mortality and incidence* of diseases used in the study and their definitions

| **Variable** | **Definition** |
| --- | --- |
| CVD mortality ^1^ | CVD was defined as diseases of the circulatory system, excluding hypertension, diseases of arteries, and lymph. The ICD-10 codes included were: I0, I11, I13, I20-I51, I60-I69. |
| MACE defitnion ^2^ | MACE was defined as CVD death or incidence of ST-elevated or non-ST elevated myocardial infarction, heart failure and stroke.  Cardiovascular mortality ICD-10: I0, I11, I13, I20-I51, I60-I69  Myocardial Infarction and heart failure ICD-10: I21, I22, I23, I24.1, I25.2, I50  Stroke ICD-10: I60, I61, I63, I64 |
| Type 2 diabetes ^3^ | ICD-10 code using death register and inpatient hospitalisation: E11.  Read Codes using general practitioner records,  Version *2*: C1041, C1096, C109A, C109B, C109C, C109E, C109F, C109G, C109H, C10F6, C10FA, C10FB, C10FC, C10FE, C10FF, C10FG, C10FH, C10FL, C10FM, C10FQ, C10FR.  Version *3*: C1011, C102, C1021, C1031, C1041, C1051, C1061, C1071, C1074, C1090, C1091, C1092, C1093, C1094, C1095, C1096, C1097, C10y1, C10z1, X40J5, X40J6, X40JJ, Xaagf, XaCJ2, XaELQ, XaEnp, XaEnq, XaF05, XaFmA, XaFn7, XaFn8, XaFn9, XaFWI, XaIrf, XaIzQ, XaIzR, XaJQp, XaKyX, XE10F, XSETH. |
| PA-related Cancer definition ^4^ | The definition of total cancer excluded in situ, benign, uncertain, non-melanoma skin cancer, or non-well-defined cancers. The ICD-10 codes used were C15, C220, C221, C34, C649, C659, C160, C54, C559, C92, C900, C18, C260, C0, C11, C12, C13, C14, C30, C31, C32, C33, C34, C38, C390, C398, C399, C199, C209, C67, C50. |

*events included fatal and nonfatal events
