# Supplementary material for: Wearable device-based health equivalence of different physical activity intensities against mortality, cardiometabolic disease, and cancer
Source: Nat Commun. 2025 Oct 7;16:8315. doi: 10.1038/s41467-025-63475-2 (PMC12504536; doi:10.1038/s41467-025-63475-2)
Supplement: Supplementary file 7 — Supplementary Data 6 [file 41467_2025_63475_MOESM7_ESM.docx]

**Supplementary Table 6:** Covariate definitions

| **Variable** | **Definition** | **UK Biobank field ID (if applicable)** |
| --- | --- | --- |
| Age | Continuous | 34, 52, accelerometer date-timestamp |
| Sex | Female/Male | 31 |
| Ethnicity | White/Others (Asia, Black, Mixed) | 21000 |
| Light intensity physical activity | Standing utilitarian movements, slow walking (<3 METs) | Derived from accelerometer data (refer to Methods) |
| Moderate intensity physical activity | Brisk walking, energetic activities (≥3 to <6 METs) | Derived from accelerometer data (refer to Methods) |
| Smoking status | Never, past, current | 20116 |
| Alcohol consumption | Never, ex-drinker, within guidelines, above guidelines | 20117, 1558 |
| Sleep duration | Hours spent sleeping | Derived from accelerometer data |
| Diet | Fruits and vegetables servings/day, categorised as low (<5 servings/day), moderate (5 to 8 servings/day) and high (>8 servings/day) | 1309, 1319, 1289, 1299 |
| Prevalent cancer | Identified by self-report and cancer registry | 20001, 100092 |
| Education | College/University; A/AS level; O levels; CSE; NVQ/HND/HNC; other | 6138 |
| Parental history of CVD | Self-reported mother or father diagnosed with heart disease or stroke | 20107, 20110 |
| Use of medication (cholesterol, blood pressure and diabetes) | Yes/No | 6177, 6153 |
| Discretionary screen time | Calculated as the sum of TV viewing time plus (non-occupational) leisure time computer use. Participants were asked: ‘In a typical day, how many hours do you spend watching TV?’ They were also asked about time spent using a computer: ‘In a typical day, how many hours do you spend using the computer? (Do not include using a computer at work)'. | 1070, 1080 |
