# Supplementary material for: Wearable device-based health equivalence of different physical activity intensities against mortality, cardiometabolic disease, and cancer
Source: Nat Commun. 2025 Oct 7;16:8315. doi: 10.1038/s41467-025-63475-2 (PMC12504536; doi:10.1038/s41467-025-63475-2)
Supplement: Supplementary file 8 — Reporting Summary [file 41467_2025_63475_MOESM8_ESM.pdf]

nature portfolio

Corresponding author(s): Emmanuel Stamatakis

Last updated by author(s): 3/03/2025

Reporting Summary

Nature Portfolio wishes to improve the reproducibility of the work that we publish. This form provides structure for consistency and transparency in reporting. For further information on Nature Portfolio policies, see our Editorial Policies and the Editorial Policy Checklist.

Statistics

For all statistical analyses, confirm that the following items are present in the figure legend, table legend, main text, or Methods section.

n/a	Confirmed	
☐	☒	The exact sample size (*n*) for each experimental group/condition, given as a discrete number and unit of measurement
☐	☒	A statement on whether measurements were taken from distinct samples or whether the same sample was measured repeatedly
☐	☒	The statistical test(s) used AND whether they are one- or two-sided<br>*Only common tests should be described solely by name; describe more complex techniques in the Methods section.*
☐	☒	A description of all covariates tested
☐	☒	A description of any assumptions or corrections, such as tests of normality and adjustment for multiple comparisons
☐	☒	A full description of the statistical parameters including central tendency (e.g. means) or other basic estimates (e.g. regression coefficient) AND variation (e.g. standard deviation) or associated estimates of uncertainty (e.g. confidence intervals)
☐	☒	For null hypothesis testing, the test statistic (e.g. *F*, *t*, *r*) with confidence intervals, effect sizes, degrees of freedom and *P* value noted<br>*Give P values as exact values whenever suitable.*
☒	☐	For Bayesian analysis, information on the choice of priors and Markov chain Monte Carlo settings
☒	☐	For hierarchical and complex designs, identification of the appropriate level for tests and full reporting of outcomes
☒	☐	Estimates of effect sizes (e.g. Cohen's *d*, Pearson's *r*), indicating how they were calculated

Our web collection on statistics for biologists contains articles on many of the points above.

Software and code

Policy information about availability of computer code

Data collection	All data was derived from the UK Biobank
Data analysis	We conducted all analyses using R statistical software (version 4.2.3), employing the RMS (version 6.3.0) and survival packages (version 3.5.5).The source data and statistical code used in the analyses of this manuscript is available upon reasonable request.

For manuscripts utilizing custom algorithms or software that are central to the research but not yet described in published literature, software must be made available to editors and reviewers. We strongly encourage code deposition in a community repository (e.g. GitHub). See the Nature Portfolio guidelines for submitting code & software for further information.

Data

Policy information about availability of data

All manuscripts must include a data availability statement. This statement should provide the following information, where applicable:

- Accession codes, unique identifiers, or web links for publicly available datasets
- A description of any restrictions on data availability
- For clinical datasets or third party data, please ensure that the statement adheres to our policy

The data that support the findings of this study are available from the UK Biobank, but restrictions apply to the availability of these data, which were used under license for the current study, and so are not publicly available. Data are however available from the authors upon reasonable request and with permission of the UK Biobank. RKB, MA and ES had full access to all the data in the study and takes responsibility for the integrity of the data and the accuracy of the data analysis. The

statistical code used in the analyses of this manuscript is available upon request. The authors have archived the statistical code of multiple similar manuscripts, e.g. https://zenodo.org/records/7187927.

Research involving human participants, their data, or biological material

Policy information about studies with human participants or human data. See also policy information about sex, gender (identity/presentation), and sexual orientation and race, ethnicity and racism.

Reporting on sex and gender	The reported sex of participants was accessed through UK Biobank data-field 31, and included as a covariate in the statistical analyses.
Reporting on race, ethnicity, or other socially relevant groupings	The participants' ethnicity was sourced from the UK Biobank data-field 21000 and incorporated as a covariate in the statistical analysis.
Population characteristics	73,485 participants with complete data were included in the study. The baseline characteristics are shown in Table 1. Core analyses were adjusted for sex, age, education, ethnicity, fruit and vegetable consumption, smoking history, alcohol consumption, sleep duration, discretionary screentime, CVD related medication use (insulin, blood pressure, cholesterol) and family history of cancer and CVD
Recruitment	The UK Biobank is a large population-based prospective cohort. Participants visited one of 22 assessment centers across England, Scotland, and Wales. 236,519 UK Biobank participants were invited to participate in an accelerometer study. From 2013 to 2015, 240,000 invitations were sent to them for PA measurement by accelerometers, with a response rate of 44%. A total of 103,684 UK Biobank participants wore a wrist-worn accelerometer on their dominant wrist for 24 hours a day over a period of 7 day. Our analysis included participants who had a minimum of 3 valid wear days (≥16 hours of wear-time per day), with at least one of those days being a weekend day. We excluded those with insufficient valid wear days, missing covariate data, or who reported an inability to walk. 73,485 participants with complete data were included in the study.
Ethics oversight	National Health Service's National Research Ethics Service in the UK (Ref: 11/NW/0382)

Note that full information on the approval of the study protocol must also be provided in the manuscript.

Field-specific reporting

Please select the one below that is the best fit for your research. If you are not sure, read the appropriate sections before making your selection.

☒ Life sciences ☐ Behavioural & social sciences ☐ Ecological, evolutionary & environmental sciences

For a reference copy of the document with all sections, see nature.com/documents/nr-reporting-summary-flat.pdf

Life sciences study design

All studies must disclose on these points even when the disclosure is negative.

Sample size	No statistical methods were used to predetermine sample size. A total of 103,684 UK Biobank participants wore a wrist-worn accelerometer on their dominant wrist for 24 hours a day over a period of 7 day. Our analysis included participants who had a minimum of 3 valid wear days (≥16 hours of wear-time per day), with at least one of those days being a weekend day. We excluded those with insufficient valid wear days, missing covariate data, or who reported an inability to walk. 73,485 participants with complete data were included in the study. At baseline, the mean age of participants was 61.6 years (SD 7.9), 41,420 (56.4%) were women and 32,065 (43.6%) were men. eFigure 1 provides a flow diagram of the study participants
Data exclusions	We excluded those with insufficient valid wear days, missing covariate data, or who reported an inability to walk. eFigure 1 provides a flow diagram of the study participants
Replication	This is a population-based cohort study, and we have not yet replicated the findings in other samples.
Randomization	This is an prospective cohort study, and randomization is not applicable in this study. However, we carefully controlled for a wide range of potential covariates in the Cox regression models to mimic randomization.
Blinding	NA

Reporting for specific materials, systems and methods

We require information from authors about some types of materials, experimental systems and methods used in many studies. Here, indicate whether each material, system or method listed is relevant to your study. If you are not sure if a list item applies to your research, read the appropriate section before selecting a response.

Materials & experimental systems

n/a	Involved in the study	
☒	☐	Antibodies
☒	☐	Eukaryotic cell lines
☒	☐	Palaeontology and archaeology
☒	☐	Animals and other organisms
☒	☐	Clinical data
☒	☐	Dual use research of concern
☒	☐	Plants

Methods

n/a	Involved in the study	
☒	☐	ChIP-seq
☒	☐	Flow cytometry
☒	☐	MRI-based neuroimaging

Plants

Seed stocks	NA
Novel plant genotypes	NA
Authentication	NA
